# Development of application and surgical technique of total knee arthroplasties: a systematic comparative analysis using worldwide registers

**DOI:** 10.1007/s00402-023-05089-1

**Published:** 2023-10-16

**Authors:** Lukas Leitner, Georg Hauer, Laura Rasic, Clemens Clar, Andreas Leithner, Patrick Sadoghi

**Affiliations:** Department of Orthopaedics and Trauma, Medical University of Graz, Auenbruggerplatz 5, 8036 Graz, Austria

**Keywords:** Total knee arthroplasty, Knee surgery, Survival curve, Arthroplasty registers

## Abstract

**Purpose:**

The aim was to compare total knee arthroplasty (TKA) between countries with regard to epidemiologic data and surgical technique by the use of worldwide registers and compare findings with a previous report after one decade.

**Methods:**

A systematic search was carried out in November 2022 and compared to previous reports from 2010. We extracted data regarding implanted TKAs, patients’ age distribution, procedure types, fixation and revision rates. After identification of 28 national arthroplasty registers, 8of them, namely Australia, Canada, Denmark, England and Wales, New Zealand, Norway, Scotland and Sweden, offered sufficient data and were included in final analysis.

**Results:**

A large variation was found in the annual number of implantations per 100,000 inhabitants with a range from 77 to 210.2 (mean 132.8). The fixation type varied strongly as well, e.g. over 95% of totally cemented TKAs in Sweden versus 61.2% cemented fixation in Denmark. Another significant difference was the use of patellar resurfacing in TKA, ranging from 47.3% (Canada) using a patellar button in TKA to less than 5% (Sweden). Within the period of one decade, the mean overall number of annual implantations increased (10%), whilst relative number of revisions decreased, the use of cemented TKA decreased from 87 to 74%, and the use of patella button remained largely inhomogeneous.

**Conclusion:**

Comparison of arthroplasty registers revealed large differences regarding the annual number of primary TKAs per inhabitant as well as primary TKA procedure types with an increase of annual implantations, decrease of relative number of revisions, and a decrease of the use of cemented systems over the period of one decade.

## Introduction

Rapid growth of joint arthroplasties numbers, beginning in the 1970s and still increasing, led to the establishment of arthroplasty registers for the sake of documentation and quality control [1, 2]. Beginning with institutional and later regional databases, the Swedish Knee Arthroplasty Register was the first full national arthroplasty register, followed by several other countries, and joints [3, 4]. Registers provide the opportunity to constantly observe the beneficial or disadvantageous effect of new materials and methods as soon as possible, and therefore allow comparative analysis of implant performance, increased revision rates and even identification of patient populations at risk for the purpose of closer follow-up [5–7]. This is in line with modern understanding of evidence-based medicine since it allows data-based continuous adaption of materials/implants and techniques to further improve patient outcome and results.

Several registers from European countries and furthermore the U.S., Canada, Australia and New Zealand have been established, and regularly publish their results, already providing an important overview and incidence, outcome, used techniques and developments in primary and revision arthroplasty of the knee [8–10]. However, no actual systematic comparisons with regard to epidemiologic data and surgical technique by the use of worldwide arthroplasty registers of total knee arthroplasty (TKA) have been published for a period of time, which allows us the comparison with a previous report after one decade [11]. This allows for the illustration of the success of total knee arthroplasty (TKA) and enables the comparison of national outcomes with one another.

The aim of this study was to compare TKA procedures between different countries regarding epidemiologic data and surgical technique using worldwide arthroplasty registers and comparing findings with a previous report after one decade.

## Materials and methods

No internal review board approval was necessary for this analysis.

The EFORT Website for European Arthroplasty Registers was used to identify the existing worldwide registers included in our study [12]. In addition, we performed a free hand search in November 2022 using the terms “(arthroplasty register) OR (knee arthroplasty register)” via Google. We focussed only on national register reports with a documentation of at least 90% of the applicable arthroplasties and/or on high-value register reports Type A.1.1.1.1 [5].

These registers were searched through to find the most recently published report from 2022 or the most recent documented time. Comparison was conducted with a previous report, using the same methodology and registers, one decade ago [11]. Three registers, namely Portugal, Spain (Catalonia) and Slovakia had to be excluded, compared to the initial report, since no actual published report was available. We extracted data with respect to the number of implanted TKAs, the patients’ age distribution, procedure types and revision rates.

These parameters were analysed for each arthroplasty register in duplicate using a qualitative content analysis of the text and compared against one another. Disagreement was resolved by discussion or, if necessary, by the decision of the senior author (PS) according to the PRISMA criteria as published by the CONSORT group [13].

The time periods considered for the number, age distribution and procedure type encompassed a duration of 1 year, with the exception of the registries of Australia and New Zealand, where data were reported for extended time periods. If included registers used different classifications with respect to age, distribution data were pooled, if applicable.

Primary total knee arthroplasty procedures were differentiated into types of procedures including total replacement with or without patellar resurfacing as well as into type of fixation including cemented, cementless, hybrid (cemented tibial and cementless femoral component) and reverse hybrid primary knee arthroplasties (cemented femoral component and cementless tibial component). In most registers, hybrid and reversed hybrid fixation procedures were not reported separately. Hybrid and reversed hybrid TKAs were, therefore, subsumed under “hybrid fixation” in the present study.

Descriptive and explorative statistical analysis as well as illustration of presented figures was performed using GraphPad PRISM 9 (GraphPad Software, San Diego, CA). As the main units of datasets were presented, no *p* values for calculation and comparison were relevant in line with previous publications in the field of arthroplasty registers [5–7, 11].

## Results

**Fig. 1:**
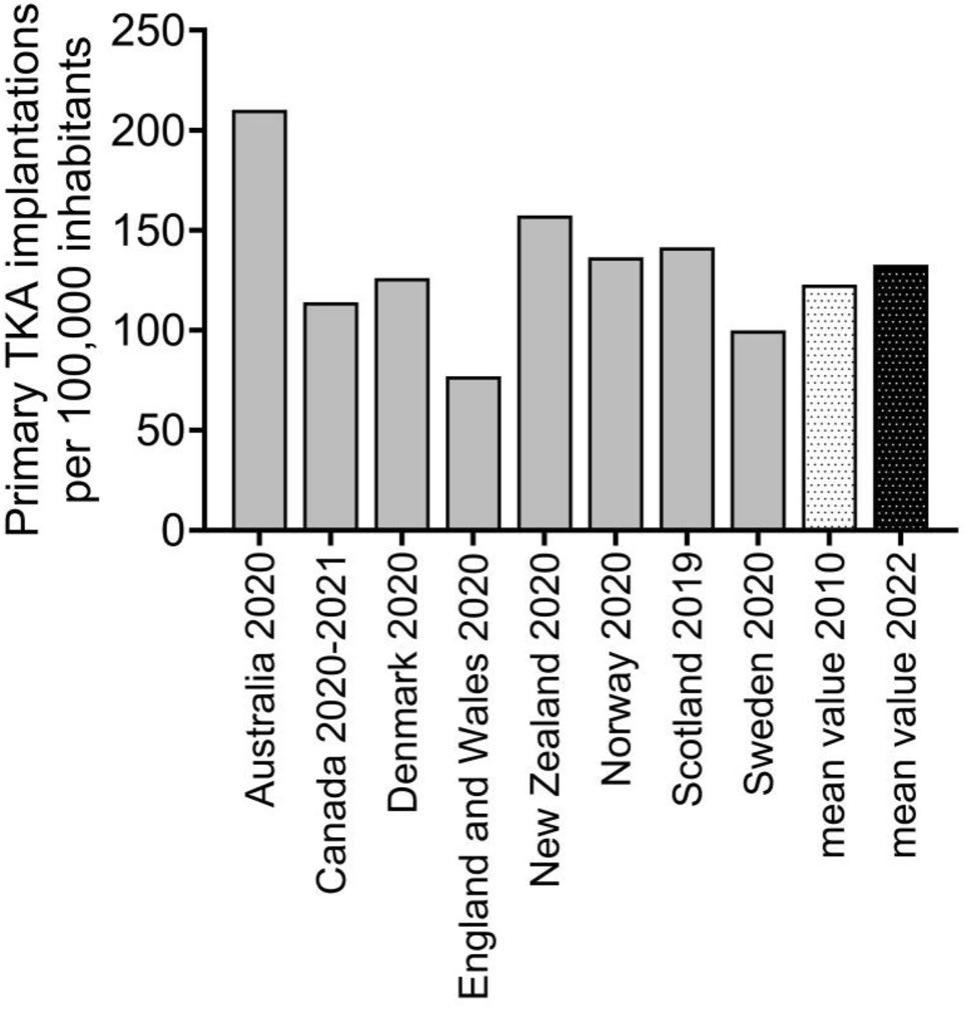
Reported number of annual primary total knee arthroplasty (TKA) implantations per inhabitant in different worldwide arthroplasty registers and mean value of all combined for this analysis from 2022

**Fig. 2:**
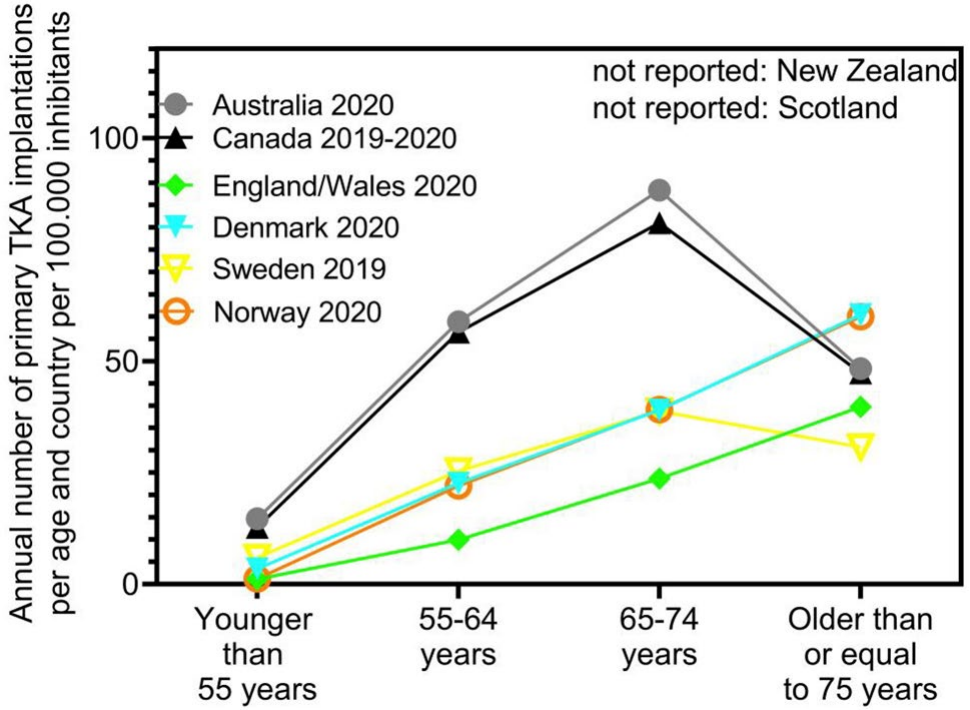
Distribution of annual primary total knee arthroplasty (TKA) implantations in worldwide arthroplasty registers according to different age groups

**Fig. 3:**
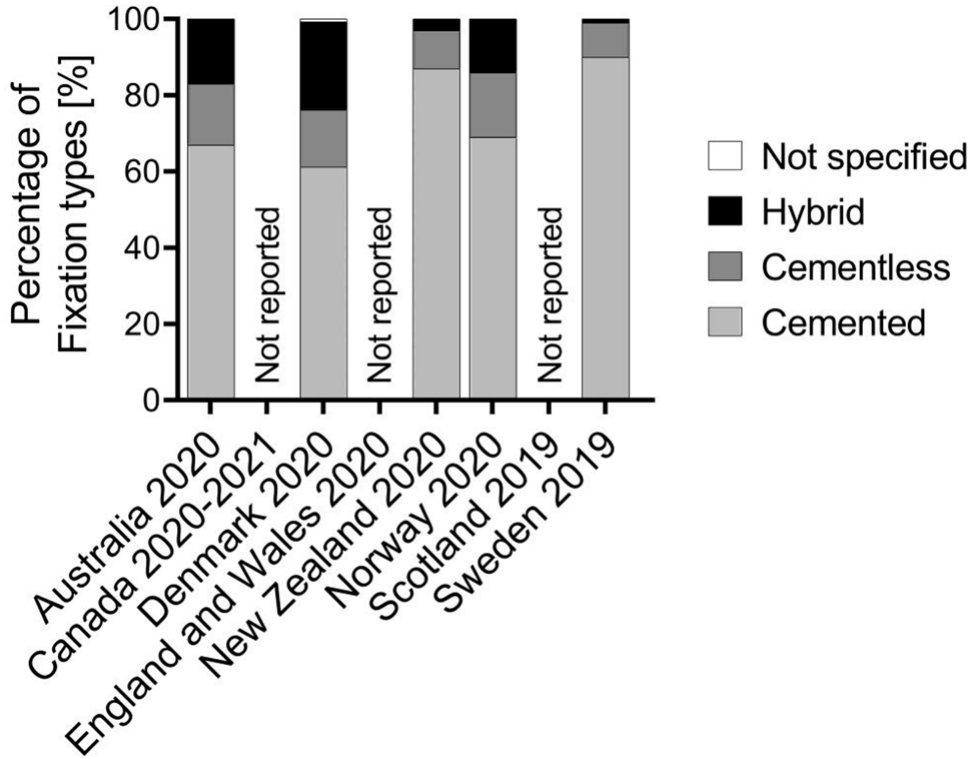
Fixation type (totally cemented, cementless or hybrid fixation) of primary total knee arthroplasty (TKA) presented in worldwide arthroplasty registers

After initial identification of 28 national or regional arthroplasty registers, 8 (England and Wales, Denmark, Norway, Sweden, Scotland, New Zealand, Australia and Canada) offered sufficient, current data and were included in final analysis. Six of the included registers are national registers, and the Scottish and English arthroplasty registers operate on regional basis. A large variation was found in the annual number of primary TKA per inhabitant with a reported range from 77 to 210 per 100,000 (mean 132.8) (Fig. 1). Compared to analysis of data from the same registers from 2013, this represents an increase of 10% (mean 122.8) [11]. Surgery was most often performed in the age groups 65–74 years and 75 years or older and least often in age group 55 years or younger in all included registers (Fig. 2). Fixation types varied strongly between the different registers, ranging from > 85% of totally cemented TKAs in Sweden and New Zealand versus 61% cemented fixation in Denmark as illustrated in Fig. 3. Compared to analysis of data from the same registers from 2013, there is clear decrease of totally cemented TKAs in most registers, and decrease in cementless fixation from 86 to 74% [11].

**Fig. 4:**
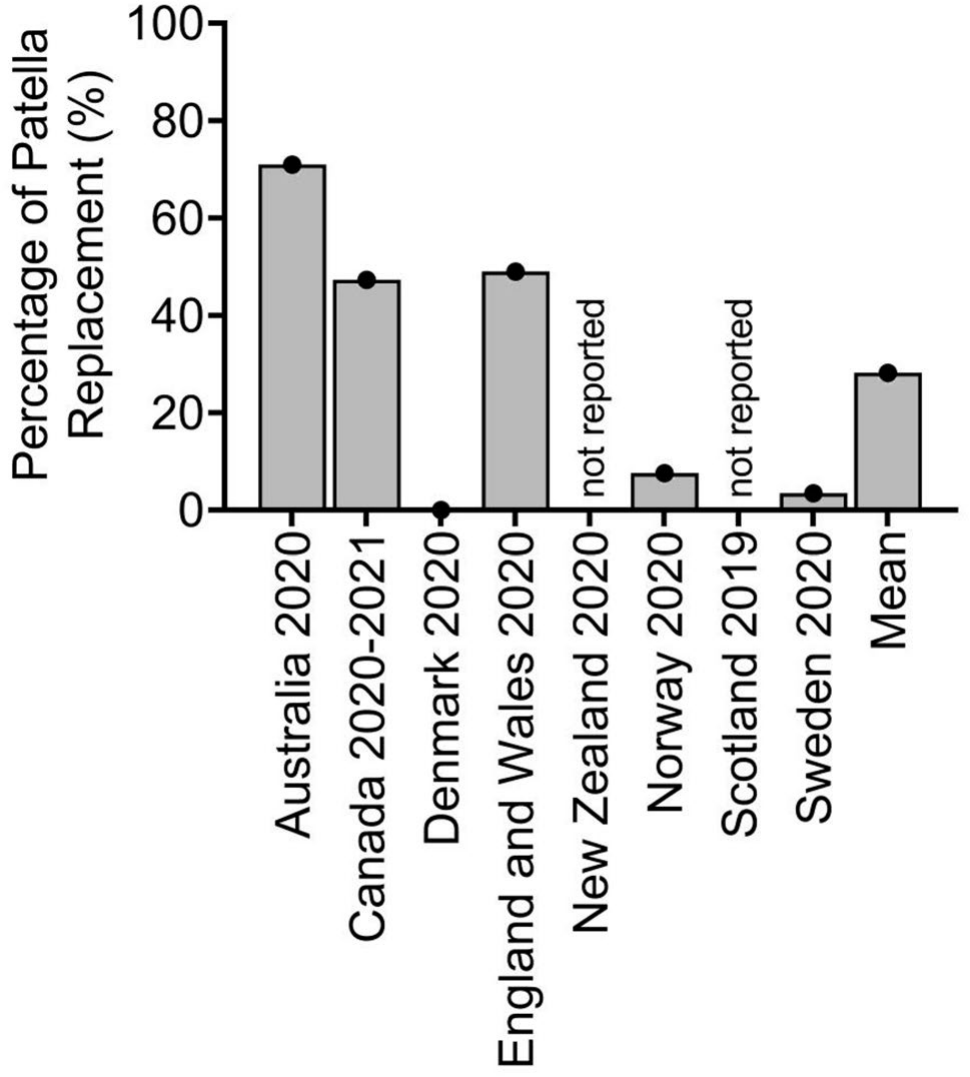
Percentage of patella replacement for primary total knee arthroplasty (TKA) in different worldwide arthroplasty registers

Another significant difference between included countries was observed with respect to the use of patellar resurfacing in TKA. Whilst the Australian knee arthroplasty register reported 71% using a patellar button in TKA in 2020, the register from Sweden reports only a minority of 4% and Denmark even 0% in the same year (Fig. 4). Compared to data from 2013, the use of patellar button in TKA in general remained on a similar level (30% compared to 31% in 2013), the most significant decrease was in Denmark (62% to 0%), whilst the most significant increase could be seen in Australia (45–71%).

Revision rates in included registries after TKA ranged from 3.5 to 13.6% (mean 6.2%), representing a decrease in five of eight included registers and a mean decrease compared to data published in 2013 (mean 8.0%) [11], as illustrated in Table 1.

**Table 1:** Number (and percentage) of annual revision rates of primary total knee arthroplasty (TKA) reported in worldwide arthroplasty register datasets, comparable data from one decade ago and direction

Register name	Absolute (relative) revisions	Published revision rate from 2013 [11]	Direction
Australia 1999–2020	22,899 (3.5%)	10.7%	--
Canada 2020–2021	4179 (7.5%)	6.3%	+
Denmark 2020	997 (13.6%)	12.5%	+
England and Wales 2020	2841 (6.2%)	6.6%	-
New Zealand 1999–2020	4421 (3.5%)	8.0%	--
Norway 2020	553 (7.7%)	9.8%	-
Scotland 2018	462 (6.3%)	8.2%	-
Sweden 2009–2018	5124 (4.1%)	3.7%	+

+ increasing, - decreasing, -- notably decreasing

## Discussion

The aim of this study was the comparison of primary total knee arthroplasty (TKA) procedures between different countries concerning epidemiologic data and surgical technique as well as revision rates using worldwide knee arthroplasty registers, and comparison to data published on the same registers a decade ago [11].

A large variation was found regarding the annual number of primary TKA implantations across the countries (Fig. 1). These variations may be explained by several factors including patient demographics (prevalence of joint disease) as well as national conditions such as healthcare systems, (insurance status), availability of performing surgeons and medical facilities and surgeon-dependent factors such as defining indications, education, tradition and experience [6, 8, 11]. Compared to data from the same registers a decade ago, in general, an increase in implantation numbers could be observed, which might be explained by ageing of population of OECD and European countries, already identified as global health challenge [14, 15]. It can be assumed that this process is ongoing, and further increasing TKA implantation numbers can be expected in developed countries [2].

Considerable differences in the proportion of fixation types between the registers could be observed. Cemented fixation is still the most used fixation, ranging from more than 90% in Sweden to less than 70% in Denmark, Australia and Norway, whilst in these countries, cementless fixation is used in over 15%, similarly hybrid in over 15%. This represents a clear increase of non-full or uncemented fixation techniques compared to one decade ago, replicating the actual trend, that cementless TKA is the subject of renewed interest [16]. This is supported by the rationale that advanced designs and modern coatings may improve biological adherence and therefore improved durability [17].

Similar to pooled outcome of register data one decade ago, this actual analysis revealed no consensus between countries with respect to the use of patellar resurfacing, with very inhomogeneous results. This observation is supported by ongoing controversial discussion in the scientific community concerning the superiority of patellar resurfacing, especially selective resurfacing [18, 19]. In this context, the Swedish register reported a higher rate of resurfaced patellae in the mid-1980s with even a lower risk of revision in case of resurfaced TKA. However, this data is biassed, because cases without patella buttons got secondary resurfaced in case of pain due to unknown causes and nowadays, TKA in Sweden is mostly performed without patella buttons [11].

In relative numbers compared to primary TKA, revision surgeries following TKA decreased in the majority of included registers within the last decade. Due to the inhomogeneity, changing frequency and interaction of patient- and implant-correlated factors leading to revision (age, sex, comorbidities) [20], we believe that it is not possible to draw further conclusions from our pooled register data.

This study has several limitations: as for any register-based analysis, results are dependent on quality of primary data, including frequently discussed difficulties, concerning comprehensiveness of included procedures, lack of standardisation of reporting age, fixation techniques or actual number of revisions [11]. Next, data on type of implant such as cruciate retaining (CR), posterior stabilised (PS) or medial pivot (MP) were missing and could not be summarised for this analysis. Last, to date, only the Swedish register reports sufficient evidence on data compliance and we cannot estimate the percentage of complete data reporting in different registers.

We want to underline the significant benefit that this study includes an analysis of pooled incidence and application type of TKA using data from the same set of worldwide arthroplasty registers over a decade.

## Conclusion

Comparison of arthroplasty registers revealed large differences regarding the annual number of primary TKAs per inhabitant as well as primary TKA procedure types with an increase of annual implantations, whilst relative number of revision surgeries decreased in most registers. We explored a decrease of the use of cemented systems over the period of one decade, in line with actual trends.
